# Psychological aging, depression, and well-being

**DOI:** 10.18632/aging.103880

**Published:** 2020-09-18

**Authors:** Maria Mitina, Sergey Young, Alex Zhavoronkov

**Affiliations:** 1 Deep Longevity, Inc., Three Exchange Square, The Landmark, Hong Kong, China; 2 Longevity Vision Fund, New York, NY 10022, USA; 3 Insilico Medicine, Hong Kong Science and Technology Park (HKSTP), Hong Kong, China; 4 The Buck Institute for Research on Aging, Novato, CA 94945, USA

**Keywords:** psychological age, subjective age, biological age, depression, well-being

## Abstract

Aging is a multifactorial process, which affects the human body on every level and results in both biological and psychological changes. Multiple studies have demonstrated that a lower subjective age is associated with better mental and physical health, cognitive functions, well-being and satisfaction with life. In this work we propose a list of non-modifiable and modifiable factors that may possibly be influenced by subjective age and its changes across an individual’s lifespan. These factors can be used for a future development of individual psychological aging clocks, which may be utilized as a sensitive measure for health status and overall life satisfaction. Furthermore, recent progress in artificial intelligence and biomarkers of biological aging have enabled scientists to discover and evaluate the efficacy of potential aging- and disease-modifying drugs and interventions. We propose that biomarkers of psychological age, which are just as important as those for biological age, may likewise be used for these purposes. Indeed, these two types of markers complement one another. We foresee the development of a broad range of parametric and deep psychological and biopsychological aging clocks, which may have implications for drug development and therapeutic interventions, and thus healthcare and other industries.

## INTRODUCTION

Like many other species, humans have a shorter lifespan in the absence of medical interventions [1]. Since the dawn of the 20^th^ century, life expectancy in developed countries has been steadily increasing primarily due to the decreases in child mortality but also due to the many advances in biotechnology and medicine [2]. Humans have had to adjust to this increase both as a society and at an individual level. Increasing life expectancy has led to substantial variability in the perception of age, as individuals may perceive themselves and others as substantially younger or older than their chronological age. The perception of subjective age may have profound effects on behavior and well-being, and is connected to an individual’s lifespan [3]. The socioemotional selectivity theory developed by Laura L. Carstensen at Stanford University, maintains that “the perception of time plays a fundamental role in the selection and pursuit of social goals” [4]. An extended perception of time may lead to knowledge-based motivations and choices. Conversely, when the perception of time is limited, a person may be motivated to preferentially make emotion-based decisions [5]. This theory and associated studies have highlighted the importance of the psychology of aging as a field and laid the foundation for studies of psychological and psychophysiological aging markers. While substantial progress has been made in identifying biomarkers of human biological aging, psychological aging is still poorly understood. There is a need for reliable tools for measuring and analyzing psychological aging, and methods for modulating longevity expectations and psychological aging states. In this paper we reflect upon the recent progress in the development of biomarkers of biological aging. We further provide a brief overview of the psychology of aging. We propose that this body of knowledge will lay the foundations for the development of next-generation biomarkers of psychological aging, dubbed psychological aging clocks, as well as deep multi-modal biopsychological and psychophysiological biomarkers of aging.

Numerous studies have demonstrated that molecular and phenotypic biomarkers may be used as effective tools for tracking healthy aging (Table 1). Since 2016, multiple deep biomarkers of aging, identified using artificial intelligence, have been proposed. These include blood biochemistry-based clocks [6], transcriptomic and proteomic aging clocks [7], epigenetic aging clocks [8], microbiome aging clocks [9], photographic aging clocks [10], and many others. These clocks may be applied very broadly to industries that are dependent on consumer health and longevity, including the pharmaceutical and consumer industries [11–13].

**Table 1:** Summary of studies based on chronological and biological age.

**Age**	**Description**	**Measures**	**References**
Chronological Age	Age in calendar years since birth.	Government-issued ID	[18]
Biological Age	Biological markers related to the state of biophysiological aging.	Molecular (based on DNA, RNA etc.)	**Methylation** [8, 19, 20]
	Commonly developed using longitudinal biological data from patients or animals in their healthy state. May be predictive of mortality, drug responses, or diseases.	Phenotypic biomarkers of aging	**Transcriptome** [7, 21]
			**Biochemistry** [6, 22, 23]
			**Microbiome** [9, 24, 25]
			**Photographs** [10]
			**Reduced representation bisulfite sequencing** [26]
			**MRI** [27]
			**EEG** [28]

These clocks can be used to assess the value of human data [14], perform data quality control [15], and many other applications. Substantial progress has been made in recent years in the use of artificial intelligence for drug discovery and biomarker development [16, 17]. Additionally, neuroimaging techniques like magnetic resonance imaging (MRI) and electroencephalogram (EEG) may be used in studies of potential biomarkers for healthy brain aging.

However, as discussed earlier, developing psychological aging clocks is also of great importance. Unlike the biological features used in biological aging clocks, many modifiable psychological aging features are easily interpretable by individuals and scientific specialists. Furthermore, methods and protocols developed for psychological age reversal may be also used in biological research for biomarker development and establishing causality. Here we review the history and state of the art in psychological aging approaches and provide a perspective on the future of psychophysiology and the psychology of aging.

### The study of psychological aging

When it comes to psychological health, a person's subjective psychological constructs may be more valuable than previously thought. Various studies have examined a number of subjective psychological concepts to understand psychological aging, including subjective age, age identity, the aging self, attitudes toward one’s own aging, self-perceptions of aging, and satisfaction with aging [29]. Historically, a single question has been used to formalize the concept of subjective age: “What age do you feel?” [30, 31] (Table 2). The answer is known as age identity, which is calculated as the difference between subjective age and chronological age [32]. Another approach for determining subjective age involves asking participants whether they feel psychologically and physically younger, older, or the same as their chronological age [33]. Further variations on this approach include asking participants to match themselves with a specific age group, such as middle-aged or older, or with a cognitive age (i.e., feel-age, look-age, do-age, and interest-age [34]. These classifications require greater implementation in longitudinal studies. In a recent study by Veenstra and colleagues [35], an analysis of longitudinal national survey data showed that a desire to be younger than one’s chronological age may be associated with lower life satisfaction and lower physical activity in the second half of a person’s life. Thus, enhanced life enjoyment is correlated with higher age satisfaction. These data raise the question of what an individual’s ideal age is, which can be interrogated by the following prompt: “If you could choose your age, what age would you like to be?” Another measure, which could be applied in clinical practice are questions about visual perceived age. This approach defines the age of participants by the perception of digital photos or physical appearance. The Longitudinal Study of Aging Danish Twins demonstrated that perceived age estimated from photographs could be used as a predictor of mortality in the volunteers. In our review we employ the term “perceived age” as related to the subjective perception of age, as opposed to visual perception [36].

**Table 2:** Summary of studies on psychological age.

**Age**	**Description**	**Measures**	**References**
Subjective Age	How does the subject feel relative to her or his chronological age?	Questions to the subject. Examples: “What age do you feel?”	[30, 31, 33, 34]
	Age is usually calculated using a survey of the individual.	“Do you feel psychologically and physically younger, the same or older than your chronological age?”	
		How participants relate themselves to a specific age group such as middle-age or older	
Ideal Age	Desire to be younger or older.	“If you could choose your age, what age would you like to be?”	[35]
Perceived visual Age	How does the subject look?	Questions about visual perception of the subject’s physical appearance.	[36]

An individual’s perceived age may influence how they overcome illness and cope with symptoms; for example, a positive view on life is linked to positive health outcomes [3]. The authors of this study further suggested that feeling younger may be an adaptive strategy in society. However, the link between mental subjective age and physiology is still not understood. Westerhof and Wurm proposed a hierarchical model that linked subjective age, psychological resources, and health. This model suggested that feeling the same as, or younger than, one’s chronological age may be associated with improved health. Alternatively, the reverse of this model may be true: better health drives a younger subjective age. In addition, the interoceptive hypothesis proposed that physical and cognitive functions decrease with age, a phenomenon that is related to an individual’s awareness of age-related changes [37, 38]. Thus, perceiving oneself as subjectively younger than one’s chronological age may influence age-related biological changes.

Numerous studies have shown that adults have a tendency to feel younger than their calendar age, and this difference increases with calendar age [39, 40]. For instance, people older than 25 years exhibited a younger subjective age [41]. In a series of studies, Weiss and colleagues also found that when older participants were confronted with negative age-related information, they perceived themselves as more similar to younger, rather than older, individuals and distanced themselves from their same-age peers [42, 43]. Studies comparing American and German populations demonstrated that adults felt younger than their calendar age, although Germans noticed an older subjective age than Americans [44]. This finding may show the youth-centeredness of American culture compared to Europe. Nevertheless, the stereotype embodiment theory [45] proposes that as adults age, they may increasingly accept society’s stereotypical expectations about their functional capacity, which in turn may influence their actual productivity and health. Thus, subjective age might depend on the socio-cultural values in a society.

Bergland and colleagues [46] demonstrated no significant differences in subjective age based on gender. However, the authors reported that men in multiple age groups (40–49 years, 50–59 years) with less education felt more youthful than those with more education. These results are, however, contradicted by previous studies where an older perceived age was correlated with fewer years of education [32, 47]. However, Kaufman and Elder [48] demonstrated that education has no significant influence on the perception of age. Accordingly, age perception may be associated with stigmatization regarding a person’s level of education and certain professional areas.

To address this issue in greater detail, we previously conducted a survey of the International Employee Benefits Association with a large, international industry group [49]. Industry professionals were employed by consulting, insurance, pension, and other companies. The surveyed individuals are experts in predicting life expectancy and mortality trends in the future. The assumptions for the mortality tables developed by the actuaries may have profound implications on insurance companies, governments, and the global economy since every extra year of an unfunded pension or a medical plan may result in billions or even trillions of dollars in liabilities. To our surprise, the longevity expectations of this group were conservative and did not account for future breakthroughs in biomedicine. This is notable, as this group of people is responsible for decisions that may affect the global economy and society. Thus, adjusting the psychological age and longevity expectations of this group of people may have a substantial positive impact.

### Subjective, chronological, and biological age

In a meta-analysis of 19 longitudinal studies, it was reported that subjective aging has a small but significant effect on health, health behaviors, and survival [3]. Stephan and colleagues showed an association between older subjective age and higher systemic inflammation and obesity [50]. Additional studies by Thyagarajan and colleagues [51] found decreased albumin concentrations in participants who felt younger. In contrast, the researchers observed higher levels of albumin in volunteers who felt older compared to a reference group. This study also showed that the prevalence of a clinically significant rise in liver enzymes, such as alanine aminotransferase, was significantly lower among the participants reporting younger subjective ages. Moreover, the researchers demonstrated that levels of cystatin C were also reduced among those who felt younger when compared with the control group. No correlations between lipids, glucose, or C-reactive protein (an inflammatory marker) and subjective age were identified. These results were partly further confirmed by Stephan and colleagues [52].

Perceived older age was also found to correlate with certain diseases, such as diabetes [53]. Moreover, subjective age was related to markers of biological age, including peak expiratory flow and grip strength [54]. Longitudinal studies have also shown that poor health, lower physical activity, body mass index, and the subjective experience of aging may be associated with cognitive abilities in later life [55].

Neurophysiology and subjective age may also be connected. For example, elderly individuals that reported a subjective age similar to or younger than their actual chronological age exhibited a higher volume of grey matter in the inferior frontal gyrus and the superior temporal gyrus; this study also found that subjective age was a predictor for younger brain age [37]. However, additional studies related to subjective age and neurophysiological mechanisms of aging are still required.

### Subjective age and stress

The central nervous system (CNS), the endocrine system and the immune system are complex and interconnected. Previous research suggested that stressful life events may negatively influence aspects of immune system function [56]. Psychological stress may increase the production of pro-inflammatory cytokines that are related to a variety of age-related diseases. For instance, catecholamines (adrenaline and noradrenaline), adrenocorticotropic hormone, cortisol, growth hormone and prolactin are all correlated with distress and adverse emotions [56]. Furthermore, age-related diseases may exacerbate the influence of stress or the effects of medical disabilities on elderly persons. Moreover, extreme stress early in life may have a long-lasting influence on the CNS, the endocrine system and the immune system.

Day-to-day variability in subjective age, such as feeling older than one’s chronological age, is associated with health issues and routine stress [57]. Indeed, researchers have suggested that everyday subjective age doesn’t vary significantly with time in the absence of other factors.

Solomon, Helvitz, and Zerach [58] showed that veterans suffering from post-traumatic stress disorder (PTSD) exhibited an older subjective age compared to veterans without PTSD. Furthermore, in a study by Palgi [59], it was demonstrated that higher levels of post-traumatic stress symptoms (PTSS) were both linearly and curvilinearly associated with a possibility of higher post-traumatic growth (PTG). PTG is defined as the positive changes that occur after trauma [60, 61]. Subjective age and perceived distance-to-death mediated this association in a linear way. Furthermore, participants who reported younger subjective age and further distance-to-death exhibited the strongest association. This was also confirmed in a previous study [62]; in contrast, the combined experience of feeling close to death and older subjective age were correlated with an increased degree of stressful events. Moreover, the effect of perceived distance-to-death on stress was softened by a perceived younger age.

In another study, ex-prisoners of war (ex-POWS) demonstrated a higher subjective age than healthy participants [63]. Additionally, ex-POWs with PTSD reported a higher subjective age than ex-POWs and volunteers without PTSD. PTSS and health measures were predictors of subjective age. Strong interactions between PTSS and health measures suggest that health only predicts subjective age in the presence of high PTSS.

These data have been corroborated by Lahav and colleagues at the molecular level by measuring telomere length, which suggested that feeling older is associated with cellular senescence [64]. Telomeres are DNA–protein complexes that cap chromosomal ends, promoting chromosomal stability. Telomeres shorten with age and thus telomere length often serves as a biomarker of cellular aging. Perceived older age was related to shorter telomeres, beyond the effect of chronological age. Variations in perceived age also mediated connections between depression and shorter telomeres.

In addition, holocaust survival and PTSD are related to attitudes toward aging and subjective age [65]. Thus, these numerous investigations show that subjective age can be used as a tool for clinical interventions in traumatized patients and for patients suffering from depressive episodes. Further investigation will be required to determine the precise interactions between these biological and psychological factors.

### Subjective age and depression

Depression is one of the most common mental illnesses worldwide. Depression may include behavioral, somatic, and cognitive impairments, and a loss of interest. Furthermore, depression can occur at any point during a human’s lifespan, and major depressive episodes (MDE) may relapse. More than half of all MDE incidents occur in individuals who experience their first MDE later in life [66]. Depression is linked to increased cortisol levels, and can thus receive negative input from the immune system. In addition, patients with depression may exhibit a perceived state of anxiety and feelings of fear [67].

Keys and Westerhof [68] have shown a link between self-perceptions of aging, chronological age, and mental health. The authors found that younger subjective age positively impacts mental health, produces a lower risk of MDE, and results in flourishing mental health (FMH). Additionally, the desire to be younger was correlated with a lower incidence of FMH and unrelated to MDE.

**Figure 1:**
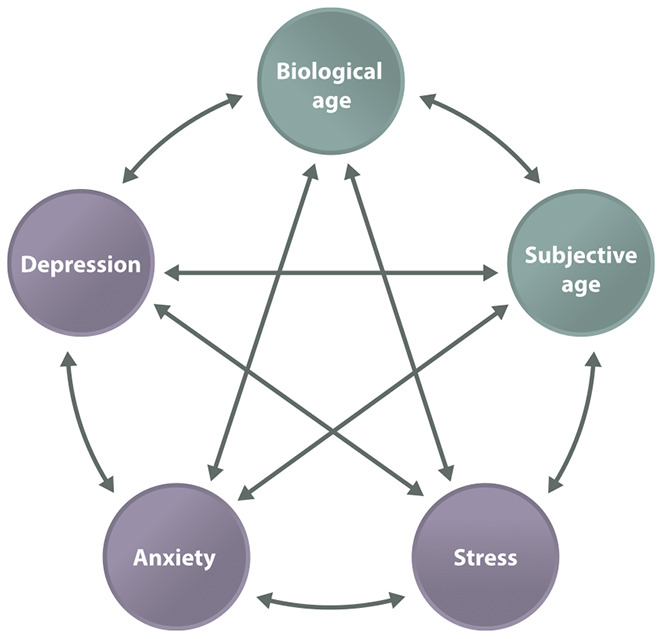
**The mind-body connection.** Biological age and subjective age are connected with a variety of diseases and may be directly linked.

In a longitudinal study by Choi and Dinitto [69], an older perceived age predicted higher depressive symptoms in the future. However, younger subjective age did not produce reduced depressive symptoms in a follow-up study. Furthermore, a longitudinal study of depression and chronic illness found that older subjective age can be a risk factor for physical morbidity and depression in the future [70]. Thus, psychological states may modulate health (Figure 1).

### Subjective age and cognitive functioning

Subjective perception of cognitive dysfunction may be associated with the early stages of dementia or morbid changes in the nervous system [37, 71, 72]. Likewise, younger subjective age is associated with better memory functioning [73]. Stephan and colleagues [55] showed that younger age feelings were associated with improved cognitive functioning 10 years later, which were determined by the strength of episodic memory and executive functioning assessments. However, this study estimated a follow-up in participants without a baseline. Furthermore, the perception of younger age was found to be related to personality traits such as openness, conscientiousness, agreeableness, and extraversion [74]. In addition to chronological age, older subjective age was correlated with a higher risk of dementia in patients over 65 during a four-year period. The authors of this study noted that this connection was caused by depressive symptoms [75]. Taken together, subjective age and cognitive abilities may be associated.

### Subjective age and mortality

Stephan and colleagues showed a relationship between subjective age and the probability of mortality in three large samples [76]. In this study, participants exhibited on average a 15% to 16% lower subjective age as compared to their calendar age. A subjective age of around 8, 11, and 13 years older in the three samples was correlated with an 18%, 29%, and 25% higher risk of mortality, respectively. These results were supported by a meta-analysis of the three samples. The authors demonstrated that chronic diseases, lack of physical activity, and cognitive issues, but not symptoms of depression, predicted the connection between subjective age and mortality. The authors concluded that a correlation exists between an older subjective age and a higher risk of mortality for adults. It was also reported that age identity could predict all-cause and cardiovascular mortality over an eight-year period. These results indicate that subjective age can be used as a biopsychosocial marker of aging. People with perceived older ages may be a potential audience for psychological interventions to modify well-being and attitudes toward aging [77].

### Subjective age and well-being

Excellent reviews on the topic of subjective well-being and related terms have been written [78, 79]. In this review we use the term “well-being” as it relates to satisfaction with life, positive and negative affect, etc. [80]. People who feel psychologically younger than their chronological age are more satisfied with their lives than those who are psychologically older [81]. Psychologically younger people have more resources, which are likely to include better mental and physical health, cognitive abilities, resilience to stress, biological age, and longevity. Mock and Eibach demonstrated that perceiving oneself as older predicts lower life satisfaction, an effect that may depend on aging attitudes [82]. They further found a relationship between higher negative affects, lower life satisfaction, and less advantageous aging attitudes.

**Figure 2:**
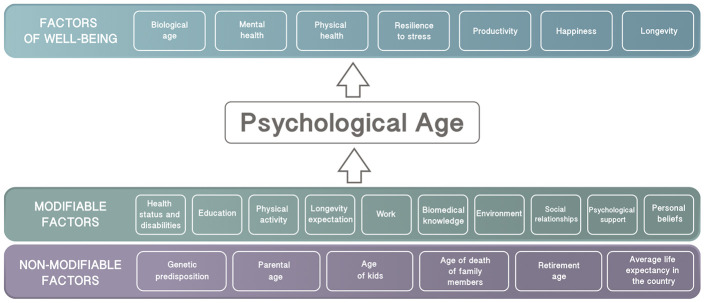
**List of modifiable and non-modifiable factors that may influence psychological age.**

There are many factors that influence psychological age and how it is related to subjective well-being (Figure 2). Some factors, so called non-modifiable factors, cannot be easily changed with behavioral modifications or therapeutic interventions. Non-modifiable factors include genetic predisposition, parental age, family members’ age of death, children’s’ age, retirement age, and average life expectancy in the country. However, there are many more factors that can be modified to reduce psychological age. These factors include health status and disabilities, physical activity, longevity expectations, education, biomedical knowledge, work, environment, psychological support, social relationships, and personal beliefs. All these factors may affect psychological age, which in turn may influence overall satisfaction with life. We propose that these modifiable factors could be used for the development of psychological aging clocks, which will require further study.

### Experimental tests of subjective age

Few studies have utilized experimental analyses to test the theoretical framework of subjective age. Stephan and colleagues showed that individuals who received positive feedback while performing a grip task experienced a younger subjective age compared to a control group without feedback [83]. In another study, Kutter-Grugn and Hess demonstrated that stereotypical negative thinking regarding age may induce older subjective age states [84]. Future work on psychological age should employ similar experimental manipulations.

Therefore, we would like to propose a series of experimental case studies to carry out in the future, in which some of the modifiable factors described in Figure 2 are manipulated in order to influence psychological age. First, we suggest that psychological affirmations could be used as an intervention to modify longevity and health expectations by 10 years. In a second case study, we could modulate participants’ responses in an experimental group by including people younger than her/him in that same group. Finally, we could design an experimental workout programme with instructions stating that the exercises would lead to feeling younger. This last experimental study was inspired by research into the placebo effect and rethinking by Alia Crum. Crum and Langer had an experimental group of hotel workers believe that their work was actually related to physical exercise and had a positive effect on health, whereas a control group of workers at the same hotel received no such instructions [85]. In this case, the experimental group showed a decrease in weight, blood pressure, waist-to-hip ratio, and body mass index after 4 weeks.

### Trends analysis and grants

**Figure 3:**
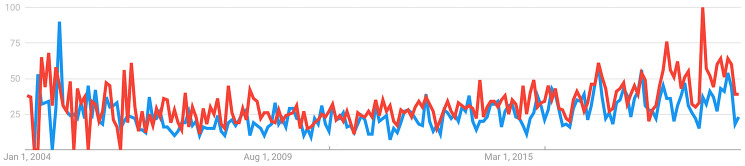
**Interest over time.** The blue line demonstrates interest in psychological age, while the red line indicates interest in biological age. The numbers represent search interest relative to the highest point on the chart over time. The value of 100 is the peak popularity for the term, while a value of 50 indicates that the term is half as popular. A score of 0 means there was not enough data for the term. Source: https://trends.google.com./

A basic search of trends analysis (Figure 3) was performed using Google Trends (https://trends.google.com/) using the keywords “psychological age” and “biological age**”.** This analysis demonstrated that, despite the increasing popularity of biological aging clocks among scientists, the topic of psychological aging is substantially less popular among the general public. Considering the link between subjective aging, health, and mental state, substantially more resources should be committed to psychological aging research. Psychological aging is just as important as biological aging and requires the development of parametric and deep psychological aging clocks to track the rate of psychological aging and to identify effective interventions to modulate psychological aging.

**Figure 4:**
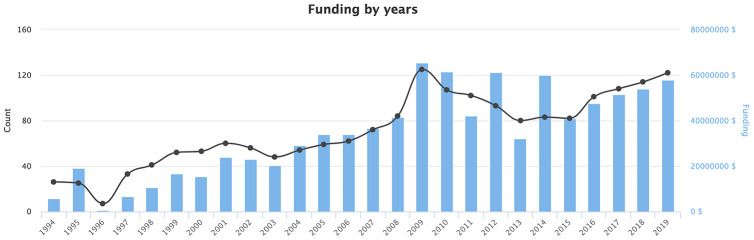
**Funding by years related to the topic of “Psychological Aging”.** Source: https://www.pharmacognitive.com/.

In addition, an analysis of the funding for psychological aging studies was performed using the open online grants search engine PharmaCognitive (http://www.pharmacognitive.com). This search engine was built using similar techniques as the International Aging Research Portfolio (IARP) [86], albeit with a significantly larger number of data sources and data types. Using the search query "Psychological Aging" (Figure 4) revealed that the amount of funding related to psychological aging research is steadily increasing and is likely to result in substantial publication activity and data availability in the coming years. The most popular topics related to funding are neurobiology of aging, psychological well-being, cognitive decline, and Alzheimer’s disease.

There is a substantial research effort directed towards the development and analysis of A National Longitudinal Study of Health & Well-Being Midlife in the United States (MIDUS, http://midus.wisc.edu/). MIDUS includes psycho-social, health, cognitive, and biomarkers measures, as well as neuroscience data (MRI, EEG). Research via PharmaCognitive showed that over 218 grants were awarded with “MIDUS” in the grant title. The majority of the grants were awarded by the National Institute on Aging (NIA) for studies supervised by Doctor Carol Ryff at the University of Wisconsin Madison, who was identified as the main Key Opinion Leader (KOL) in the field. In addition, there were more than 300 publications with “MIDUS” as a keyword. In addition to MIDUS, similar studies have been carried out in other countries, such as Midlife in Japan (MIDJA). The principal aim was to compare MIDJA with MIDUS to investigate the influence of psychosocial factors on the health of mid- and later-life adults in Japan and the United States.

In addition, the Leipzig Study for Mind-Body-Emotion Interactions (LEMON, http://fcon_1000.projects.nitrc.org/indi/retro/MPI_LEMON.html) features datasets for healthy participants from a number of different age groups [87]. LEMON is a part of the larger Max Planck Institute Leipzig Mind-Brain-Body database, and contains psychological and physiological data, including EEG and MRI measures. There is a similar public resource for data on aging in America, which has existed since 1900, called the Health Retirement Study (HRS, https://hrs.isr.umich.edu/). This study includes data on cognition, health, psycho-social, biomarkers, and genetic data. There are more than 5000 publications related to HRS.

## DISCUSSION

As indicated in this review, a broad literature suggests that there is a relationship between age identity and health, mental states, cognitive functioning, longevity, and well-being. Increasing human productive longevity by slowing down or even reversing biological and psychological aging will help accelerate economic growth in major developed countries [88, 89]. Subjective aging is determined by various parameters such as health changes, personal experiences, social relationships, and cultural values. Given the strong connection between aging and general factors of well-being, promoting a positive attitude towards one’s own aging may be an important aim for public health efforts and clinics.

Despite numerous studies on subjective age, only a limited number of related biomarkers have been examined. For instance, the combined influence of perceived subjective age, epigenetic factors, and biological systems, such as the central nervous system, peripheral system, and immune system, will require more precise research. A complex approach may shed light on age-related changes and the risk of future mental illnesses, which can additionally be associated with productive functioning.

Multiple studies have demonstrated that lower subjective age is associated with better mental and physical health, cognitive functions, and satisfaction with life. The ability to precisely measure subjective or psychological age and identify the key modifiable factors, evaluate their importance, and analyze the correlations between these factors may help improve the quality of life of patients and the general population. Future investigations are needed to further contribute to the understanding of the practical implementation of such measures. In this review we also propose a list of non-modifiable and modifiable factors, which may be influenced by subjective age and its changes across an individual’s lifespan. We intend to use these modifiable psychological factors, in combination with biological factors, as important features for the development of psychological aging clocks.

In addition, in order to increase individuals’ resilience to stress and achieve positive behavioral changes, new tools for evaluating biopsychological profiles should be developed. Recent advances in artificial intelligence allow for the development of multi-modal biomarkers of aging. However, the majority of these efforts are focused on biological aging clocks. We speculate that the development of psychological aging clocks using deep learning techniques may be just as impactful and may help validate and improve these deep learning approaches, as psychological survey, lifestyle, and behavioral data is usually more interpretable. We foresee the development of many types of deep psychological, psychophysiological, and biopsychological aging clocks using machine learning techniques, and believe they may one day be used as standard tools in psychiatry, longevity research, and in a broad range of applications across many industries.

## References

[1] Tidière M, Gaillard JM, Berger V, Müller DW, Bingaman Lackey L, Gimenez O, Clauss M, Lemaître JF. Comparative analyses of longevity and senescence reveal variable survival benefits of living in zoos across mammals. Sci Rep. 2016; 6:36361. 10.1038/srep36361 27819303 PMC5098244

[2] Mathers CD, Stevens GA, Boerma T, White RA, Tobias MI. Causes of international increases in older age life expectancy. Lancet. 2015; 385:540–48. 10.1016/S0140-6736(14)60569-9 25468166

[3] Westerhof GJ, Wurm S. Longitudinal research on subjective aging, health, and longevity: Current evidence and new directions for research. Annual Review of Gerontology and Geriatrics. 2015; 35:145–65. 10.1891/0198-8794.35.145

[4] Carstensen LL, Isaacowitz DM, Charles ST. Taking time seriously. A theory of socioemotional selectivity. Am Psychol. 1999; 54:165–81. 10.1037//0003-066x.54.3.165 10199217

[5] Carstensen LL. The influence of a sense of time on human development. Science. 2006; 312:1913–15. 10.1126/science.1127488 16809530 PMC2790864

[6] Mamoshina P, Kochetov K, Cortese F, Kovalchuk A, Aliper A, Putin E, Scheibye-Knudsen M, Cantor CR, Skjodt NM, Kovalchuk O, Zhavoronkov A. Blood biochemistry analysis to detect smoking status and quantify accelerated aging in smokers. Sci Rep. 2019; 9:142. 10.1038/s41598-018-35704-w 30644411 PMC6333803

[7] Mamoshina P, Volosnikova M, Ozerov IV, Putin E, Skibina E, Cortese F, Zhavoronkov A. Machine learning on human muscle transcriptomic data for biomarker discovery and tissue-specific drug target identification. Front Genet. 2018; 9:242. 10.3389/fgene.2018.00242 30050560 PMC6052089

[8] Moskalev A, Anisimov V, Aliper A, Artemov A, Asadullah K, Belsky D, Baranova A, de Grey A, Dixit VD, Debonneuil E, Dobrovolskaya E, Fedichev P, Fedintsev A, et al A review of the biomedical innovations for healthy longevity. Aging (Albany NY). 2017; 9:7–25. 10.18632/aging.101163

[9] Galkin F, Mamoshina P, Aliper A, Putin E, Moskalev V, Gladyshev VN, Zhavoronkov A. Human gut microbiome aging clock based on taxonomic profiling and deep learning. iScience. 2020; 23:101199. 10.1016/j.isci.2020.101199 32534441 PMC7298543

[10] Bobrov E, Georgievskaya A, Kiselev K, Sevastopolsky A, Zhavoronkov A, Gurov S, Rudakov K, Del Pilar Bonilla Tobar M, Jaspers S, Clemann S. PhotoAgeClock: deep learning algorithms for development of non-invasive visual biomarkers of aging. Aging (Albany NY). 2018; 10:3249–3259. 10.18632/aging.101629 30414596 PMC6286834

[11] Galkin F, Mamoshina P, Aliper A, de Magalhães JP, Gladyshev VN, Zhavoronkov A. Biohorology and biomarkers of aging: current state-of-the-art, challenges and opportunities. Ageing Res Rev. 2020; 60:101050. 10.1016/j.arr.2020.101050 32272169

[12] Mamoshina P, Zhavoronkov A. Deep Integrated Biomarkers of Aging. In Biomarkers of Human Aging. 2019; 281–91. 10.1007/978-3-030-24970-0_18

[13] Zhavoronkov A, Li R, Ma C, Mamoshina P. Deep biomarkers of aging and longevity: from research to applications. Aging (Albany NY). 2019; 11:10771–80. 10.18632/aging.102475 31767810 PMC6914424

[14] Zhavoronkov A, Church G. The advent of human life data economics. Trends Mol Med. 2019; 25:566–70. 10.1016/j.molmed.2019.05.002 31257078

[15] Mamoshina P, Kochetov K, Putin E, Aliper A. Testing for batch effect through age predictors Data. bioRxiv. 2019; 1–10. 10.1101/531863

[16] Zhavoronkov A. Artificial Intelligence for Drug Discovery, Biomarker Development, and Generation of Novel Chemistry. Mol Pharm. 2018; 15:4311–4313. 10.1021/acs.molpharmaceut.8b00930 30269508

[17] Zhavoronkov A, Mamoshina P, Vanhaelen Q, Scheibye-Knudsen M, Moskalev A, Aliper A. Artificial intelligence for aging and longevity research: recent advances and perspectives. Ageing Res Rev. 2019; 49:49–66. 10.1016/j.arr.2018.11.003 30472217

[18] Kent EM. Oxford Dictionary of Sports Science and Medicine. J Sports Sci Med. 2007; 6:152.

[19] Hannum G, Guinney J, Zhao L, Zhang L, Hughes G, Sadda S, Klotzle B, Bibikova M, Fan JB, Gao Y, Deconde R, Chen M, Rajapakse I, et al. Genome-wide methylation profiles reveal quantitative views of human aging rates. Mol Cell. 2013; 49:359–67. 10.1016/j.molcel.2012.10.016 23177740 PMC3780611

[20] Horvath S. DNA methylation age of human tissues and cell types. Genome Biol. 2013; 14:R115. 10.1186/gb-2013-14-10-r115 24138928 PMC4015143

[21] Peters MJ, Joehanes R, Pilling LC, Schurmann C, Conneely KN, Powell J, Reinmaa E, Sutphin GL, Zhernakova A, Schramm K, Wilson YA, Kobes S, Tukiainen T, et al, and NABEC/UKBEC Consortium. The transcriptional landscape of age in human peripheral blood. Nat Commun. 2015; 6:8570. 10.1038/ncomms9570 26490707 PMC4639797

[22] Putin E, Mamoshina P, Aliper A, Korzinkin M, Moskalev A, Kolosov A, Ostrovskiy A, Cantor C, Vijg J, Zhavoronkov A. Deep biomarkers of human aging: application of deep neural networks to biomarker development. Aging (Albany NY). 2016; 8:1021–33. 10.18632/aging.100968 27191382 PMC4931851

[23] Mamoshina P, Kochetov K, Putin E, Cortese F, Aliper A, Lee WS, Ahn SM, Uhn L, Skjodt N, Kovalchuk O, Scheibye-Knudsen M, Zhavoronkov A. Population specific biomarkers of human aging: a big data study using South Korean, Canadian, and Eastern European patient populations. J Gerontol A Biol Sci Med Sci. 2018; 73:1482–90. 10.1093/gerona/gly005 29340580 PMC6175034

[24] Galkin F, Aliper A, Putin E, Kuznetsov I, Gladyshev VN, Zhavoronkov A. Human microbiome aging clocks based on deep learning and tandem of permutation feature importance and accumulated local effects. bioRxiv. 2018 10.1101/507780

[25] Hertel J, Friedrich N, Wittfeld K, Pietzner M, Budde K, Van der Auwera S, Lohmann T, Teumer A, Völzke H, Nauck M, Grabe HJ. Measuring biological age via metabonomics: the metabolic age score. J Proteome Res. 2016; 15:400–10. 10.1021/acs.jproteome.5b00561 26652958

[26] Petkovich DA, Podolskiy DI, Lobanov AV, Lee SG, Miller RA, Gladyshev VN. Using DNA methylation profiling to evaluate biological age and longevity interventions. Cell Metab. 2017; 25:954–60.e6. 10.1016/j.cmet.2017.03.016 28380383 PMC5578459

[27] Franke K, Luders E, May A, Wilke M, Gaser C. Brain maturation: predicting individual BrainAGE in children and adolescents using structural MRI. Neuroimage. 2012; 63:1305–12. 10.1016/j.neuroimage.2012.08.001 22902922

[28] Sun H, Paixao L, Oliva JT, Goparaju B, Carvalho DZ, van Leeuwen KG, Akeju O, Thomas RJ, Cash SS, Bianchi MT, Westover MB. Brain age from the electroencephalogram of sleep. Neurobiol Aging. 2019; 74:112–20. 10.1016/j.neurobiolaging.2018.10.016 30448611 PMC6478501

[29] Diehl M, Wahl HW, Barrett AE, Brothers AF, Miche M, Montepare JM, Westerhof GJ, Wurm S. Awareness of aging: theoretical considerations on an emerging concept. Dev Rev. 2014; 34:93–113. 10.1016/j.dr.2014.01.001 24958998 PMC4064469

[30] Barak B, Stern B. Subjective age correlates: a research note. Gerontologist. 1986; 26:571–78. 10.1093/geront/26.5.571 3533727

[31] Peters GR. Self-conceptions of the aged, age identification, and aging. Gerontologist. 1971; 11:69–73 10.1093/geront/11.4_part_2.69 5118193

[32] Barrett AE. Socioeconomic status and age identity: the role of dimensions of health in the subjective construction of age. J Gerontol B Psychol Sci Soc Sci. 2003; 58:S101–09. 10.1093/geronb/58.2.s101 12646599

[33] Uotinen V, Rantanen T, Suutama T. Perceived age as a predictor of old age mortality: a 13-year prospective study. Age Ageing. 2005; 34:368–72. 10.1093/ageing/afi091 15899910

[34] Kastenbaum R, Derbin V, Sabatini P, Artt S. “The Ages of Me”: Toward Personal and Interpersonal Definitions of Functional Aging. Aging Hum Dev. (Los Angeles, CA). 1972; 3:197–211. 10.2190/TUJR-WTXK-866Q-8QU7

[35] Veenstra M, Daatland SO, Aartsen M. The role of subjective age in sustaining wellbeing and health in the second half of life. Ageing Soc. 2020; 1–21. 10.1017/S0144686X2000032X

[36] Christensen K, Iachina M, Rexbye H, Tomassini C, Frederiksen H, McGue M, Vaupel JW. “looking old for your age”: genetics and mortality. Epidemiology. 2004; 15:251–52. 10.1097/01.ede.0000112211.11416.a6 15127920

[37] Kwak S, Kim H, Chey J, Youm Y. Feeling how old I am: subjective age is associated with estimated brain age. Front Aging Neurosci. 2018; 10:168. 10.3389/fnagi.2018.00168 29930506 PMC5999722

[38] Diehl MK, Wahl HW. Awareness of age-related change: examination of a (mostly) unexplored concept. J Gerontol B Psychol Sci Soc Sci. 2010; 65B:340–50. 10.1093/geronb/gbp110 20008026 PMC2853600

[39] Goldsmith RE, Heiens RA. Subjective age: a test of five hypotheses. Gerontologist. 1992; 32:312–17. 10.1093/geront/32.3.312 1499995

[40] Montepare JM, Lachman ME. “you’re only as old as you feel”: self-perceptions of age, fears of aging, and life satisfaction from adolescence to old age. Psychol Aging. 1989; 4:73–78. 10.1037//0882-7974.4.1.73 2803614

[41] Rubin DC, Berntsen D. People over forty feel 20% younger than their age: subjective age across the lifespan. Psychon Bull Rev. 2006; 13:776–80. 10.3758/bf03193996 17328372 PMC3969748

[42] Weiss D, Freund AM. Still young at heart: negative age-related information motivates distancing from same-aged people. Psychol Aging. 2012; 27:173–80. 10.1037/a0024819 21823797

[43] Weiss D, Lang FR. “they” are old but “I” feel younger: age-group dissociation as a self-protective strategy in old age. Psychol Aging. 2012; 27:153–63. 10.1037/a0024887 21988154

[44] Westerhof G. Forever young? A comparison of age identities in the United States and Germany. Res Aging. 2003; 25:366–83. 10.1177/0164027503025004002

[45] Levy B. Stereotype embodiment: a psychosocial approach to aging. Curr Dir Psychol Sci. 2009; 18:332–36. 10.1111/j.1467-8721.2009.01662.x 20802838 PMC2927354

[46] Bergland A, Nicolaisen M, Thorsen K. Predictors of subjective age in people aged 40-79 years: a five-year follow-up study. The impact of mastery, mental and physical health. Aging Ment Health. 2014; 18:653–61. 10.1080/13607863.2013.869545 24359016

[47] Markides KS, Boldt JS. Change in subjective age among the elderly: a longitudinal analysis. Gerontologist. 1983; 23:422–27. 10.1093/geront/23.4.422 6604681

[48] Kaufman G, Elder GH. Grandparenting and age identity. J Aging Stud. 2003; 17:269–82. 10.1016/S0890-4065(03)00030-6

[49] Zhavoronkov A. Longevity expectations in the pension fund, insurance, and employee benefits industries. Psychol Res Behav Manag. 2015; 8:27–39. 10.2147/PRBM.S75440 25653568 PMC4309776

[50] Stephan Y, Sutin AR, Terracciano A. Subjective age and adiposity: evidence from five samples. International J of Obesity. 2019; 4:938–41. 10.1038/s41366-018-0179-x 30250240

[51] Thyagarajan B, Shippee N, Parsons H, Vivek S, Crimmins E, Faul J, Shippee T. How does subjective age get “under the skin”? the association between biomarkers and feeling older or younger than one’s age: the health and retirement study. Innov Aging. 2019; 3:igz035. 10.1093/geroni/igz035 31528718 PMC6736363

[52] Stephan Y, Sutin AR, Terracciano A. Subjective age and cystatin C among older adults. J Gerontol B Psychol Sci Soc Sci. 2019; 74:382–88. 10.1093/geronb/gbx124 29045722 PMC6377033

[53] Demakakos P, Gjonca E, Nazroo J. Age identity, age perceptions, and health: evidence from the english longitudinal study of ageing. Ann N Y Acad Sci. 2007; 1114:279–87. 10.1196/annals.1396.021 17986588

[54] Stephan Y, Sutin AR, Terracciano A. How old do you feel? the role of age discrimination and biological aging in subjective age. PLoS One. 2015; 10:e0119293. 10.1371/journal.pone.0119293 25738579 PMC4349738

[55] Stephan Y, Caudroit J, Jaconelli A, Terracciano A. Subjective age and cognitive functioning: a 10-year prospective study. Am J Geriatr Psychiatry. 2014; 22:1180–87. 10.1016/j.jagp.2013.03.007 23871114

[56] Glaser R, Kiecolt-Glaser JK. Stress-induced immune dysfunction: implications for health. Nat Rev Immunol. 2005; 5:243–51. 10.1038/nri1571 15738954

[57] Kotter-Grühn D, Neupert SD, Stephan Y. Feeling old today? daily health, stressors, and affect explain day-to-day variability in subjective age. Psychol Health. 2015; 30:1470–85. 10.1080/08870446.2015.1061130 26066614

[58] Solomon Z, Helvitz H, Zerach G. Subjective age, PTSD and physical health among war veterans. Aging Ment Health. 2009; 13:405–13. 10.1080/13607860802459856 19484604

[59] Palgi Y. Subjective age and perceived distance-to-death moderate the association between posttraumatic stress symptoms and posttraumatic growth among older adults. Aging Ment Health. 2016; 20:948–54. 10.1080/13607863.2015.1047320 26028224

[60] Joseph S, Linley PA. Trauma, Recovery, and Growth: Positive Psychological Perspectives on Posttraumatic Stress. John Wiley & Sons. 2008 10.1002/9781118269718

[61] Calhoun L, & Tedeschi R. Posttraumatic growth in clinical practice. Routled. 2012 10.4324/9780203629048

[62] Shrira A, Bodner E, Palgi Y. The interactive effect of subjective age and subjective distance-to-death on psychological distress of older adults. Aging Ment Health. 2014; 18:1066–70. 10.1080/13607863.2014.915925 24831662

[63] Avidor S, Benyamini Y, Solomon Z. Subjective age and health in later life: the role of posttraumatic symptoms. J Gerontol B Psychol Sci Soc Sci. 2016; 71:415–24. 10.1093/geronb/gbu150 25324296

[64] Lahav Y, Avidor S, Stein JY, Zhou X, Solomon Z. Telomere length and depression among ex-prisoners of war: the role of subjective age. J Gerontol B Psychol Sci Soc Sci. 2020; 75:21–29. 10.1093/geronb/gby006 29415270

[65] Shrira A. Parental holocaust exposure, related PTSD symptoms and subjective aging across the generations. J Gerontol B Psychol Sci Soc Sci. 2020; 75:30–41. 10.1093/geronb/gbz012 30690549

[66] Fiske A, Wetherell JL, Gatz M. Depression in older adults. Annu Rev Clin Psychol. 2009; 5:363–89. 10.1146/annurev.clinpsy.032408.153621 19327033 PMC2852580

[67] Lovibond PF, Lovibond SH. The structure of negative emotional states: comparison of the depression anxiety stress scales (DASS) with the beck depression and anxiety inventories. Behav Res Ther. 1995; 33:335–43. 10.1016/0005-7967(94)00075-u 7726811

[68] Keyes CL, Westerhof GJ. Chronological and subjective age differences in flourishing mental health and major depressive episode. Aging Ment Health. 2012; 16:67–74. 10.1080/13607863.2011.596811 21780972

[69] Choi NG, DiNitto DM. Felt age and cognitive-affective depressive symptoms in late life. Aging Ment Health. 2014; 18:833–37. 10.1080/13607863.2014.886669 24533681 PMC4349513

[70] Segel-Karpas D, Palgi Y, Shrira A. The reciprocal relationship between depression and physical morbidity: the role of subjective age. Health Psychol. 2017; 36:848–51. 10.1037/hea0000542 28737414 PMC5666207

[71] Reid LM, Maclullich AM. Subjective memory complaints and cognitive impairment in older people. Dement Geriatr Cogn Disord. 2006; 22:471–85. 10.1159/000096295 17047326

[72] Yasuno F, Kazui H, Yamamoto A, Morita N, Kajimoto K, Ihara M, Taguchi A, Matsuoka K, Kosaka J, Tanaka T, Kudo T, Takeda M, Nagatsuka K, et al. Resting-state synchrony between the retrosplenial cortex and anterior medial cortical structures relates to memory complaints in subjective cognitive impairment. Neurobiol Aging. 2015; 36:2145–52. 10.1016/j.neurobiolaging.2015.03.006 25862421

[73] Stephan Y, Sutin AR, Caudroit J, Terracciano A. Subjective age and changes in memory in older adults. J Gerontol B Psychol Sci Soc Sci. 2016; 71:675–83. 10.1093/geronb/gbv010 25748213

[74] Stephan Y, Sutin AR, Terracciano A. Subjective age and personality development: a 10-year study. J Pers. 2015; 83:142–54. 10.1111/jopy.12090 24471687

[75] Stephan Y, Sutin AR, Luchetti M, Terracciano A. Subjective age and risk of incident dementia: evidence from the national health and aging trends survey. J Psychiatr Res. 2018; 100:1–4. 10.1016/j.jpsychires.2018.02.008 29471080 PMC5866231

[76] Stephan Y, Sutin AR, Terracciano A. Subjective age and mortality in three longitudinal samples. Psychosom Med. 2018; 80:659–64. 10.1097/PSY.0000000000000613 29864106 PMC6345273

[77] Rippon I, Steptoe A. Feeling old vs being old: associations between self-perceived age and mortality. JAMA Intern Med. 2015; 175:307–09. 10.1001/jamainternmed.2014.6580 25506678

[78] Chekola M. The concept of happiness. 1974; Doctoral dissertation, University of Michigan.

[79] Diener E. Subjective Well-Being. Springer, Dordrecht; 2009 11–58. 10.1007/978-90-481-2350-6_2

[80] Andrews FM, Withey SB, Andrews FM, Withey SB. Measuring Global Well-Being. Social Indicators of Well-Being. Springer US; 1976 63–106. 10.1007/978-1-4684-2253-5_3

[81] Stephan Y, Sutin AR, Terracciano A. Determinants and Implications of Subjective Age Across Adulthood and Old Age. The Oxford handbook of integrative health science. 2018; 3:87–96. 10.1093/oxfordhb/9780190676384.013.7

[82] Mock SE, Eibach RP. Aging attitudes moderate the effect of subjective age on psychological well-being: evidence from a 10-year longitudinal study. Psychol Aging. 2011; 26:979–86. 10.1037/a0023877 21728444

[83] Stephan Y, Chalabaev A, Kotter-Grühn D, Jaconelli A. "Feeling younger, being stronger": an experimental study of subjective age and physical functioning among older adults. J Gerontol B Psychol Sci Soc Sci. 2013; 68:1–7. 10.1093/geronb/gbs037 22492113

[84] Kotter-Grühn D, Hess TM. The impact of age stereotypes on self-perceptions of aging across the adult lifespan. J Gerontol B Psychol Sci Soc Sci. 2012; 67:563–71. 10.1093/geronb/gbr153 22367710 PMC3441190

[85] Crum AJ, Langer EJ. Mind-set matters: exercise and the placebo effect. Psychol Sci. 2007; 18:165–71. 10.1111/j.1467-9280.2007.01867.x 17425538

[86] Zhavoronkov A, Cantor CR. Methods for structuring scientific knowledge from many areas related to aging research. PLoS One. 2011; 6:e22597. 10.1371/journal.pone.0022597 21799912 PMC3142169

[87] Babayan A, Erbey M, Kumral D, Reinelt JD, Reiter AM, Röbbig J, Schaare HL, Uhlig M, Anwander A, Bazin PL, Horstmann A, Lampe L, Nikulin VV, et al. A mind-brain-body dataset of MRI, EEG, cognition, emotion, and peripheral physiology in young and old adults. Sci Data. 2019; 6:180308. 10.1038/sdata.2018.308 30747911 PMC6371893

[88] Zhavoronkov A, The ageless generation: How advances in biomedicine will transform the global economy. St. Martin's Press 2013.

[89] Zhavoronkov A, Litovchenko M. Biomedical progress rates as new parameters for models of economic growth in developed countries. Int J Environ Res Public Health. 2013; 10:5936–52. 10.3390/ijerph10115936 24217179 PMC3863879

